# Combined Phosphoproteomics and Bioinformatics Strategy in Deciphering Drug Resistant Related Pathways in Triple Negative Breast Cancer

**DOI:** 10.1155/2014/390781

**Published:** 2014-11-13

**Authors:** Xinyu Deng, Morris Kohanfars, Huan Ming Hsu, Puneet Souda, Joe Capri, Julian P. Whitelegge, Helena R. Chang

**Affiliations:** ^1^Gonda/UCLA Breast Cancer Research Laboratory and the Revlon/UCLA Breast Center, Department of Surgery, David Geffen School of Medicine, University of California at Los Angeles, 200 Med Plaza, Ste B265-1, Los Angeles, CA 90095-7028, USA; ^2^The Pasarow Mass Spectrometry Laboratory, NPI-Semel Institute, University of California at Los Angeles, Los Angeles, CA, USA

## Abstract

Because of the absence of a clear therapeutic target for triple negative breast cancer (TNBC), conventional chemotherapy is the only available systemic treatment option for these patients. Despite chemotherapy treatment, TNBC patients still have worse prognosis when compared with other breast cancer patients. The study is to investigate unique phosphorylated proteins expressed in chemoresistant TNBC cell lines. In the current study, twelve TNBC cell lines were subjected to drug sensitivity assays against chemotherapy drugs docetaxel, doxorubicin, gemcitabine, and cisplatin. Based on their half maximal inhibitory concentrations, four resistant and two sensitive cell lines were selected for further analysis. The phosphopeptides from these cells were enriched with TiO_2_ beads and fractionated using strong cation exchange. 1,645 phosphoprotein groups and 9,585 unique phosphopeptides were identified by a high throughput LC-MS/MS system LTQ-Orbitrap. The phosphopeptides were further filtered with Ascore system and 1,340 phosphoprotein groups, 2,760 unique phosphopeptides, and 4,549 unique phosphosites were identified. Our study suggested that differentially phosphorylated Cdk5, PML, AP-1, and HSF-1 might work together to promote vimentin induced epithelial to mesenchymal transition (EMT) in the drug resistant cells. EGFR and HGF were also shown to be involved in this process.

## 1. Introduction

Breast cancer is the most common cancer in women [1]. Although the overall incidence of breast cancer is rising worldwide, the mortality rate has been decreasing in the United States [2]. The improved survival rate is likely to be a result of the success in early detection and better treatment in patients with positive estrogen receptors (ER), progesterone receptors (PR), or human epidermal growth factor receptor 2 (Her2/neu) breast cancers [3]. Triple negative breast cancers (TNBC) by default have been grouped together because of the lack of ER, PR, and Her2/neu markers [4, 5]. Compared to the other subtypes of breast cancer, these tumors are frequently more aggressive, manifested by a higher distant relapse rate with more frequent visceral as well as central nervous system metastases and higher mortality rate despite chemotherapy [6–8]. The heterogeneous biology and histopathology of TNBC underlie the unpredictable responses to chemotherapy and diverse clinical outcomes seen in these patients. The majority of TNBC with relapse is multidrug resistant and ultimately becomes refractory to all therapies [9, 10]. To improve treatment, it is important to develop novel therapeutic strategies to predict and overcome drug resistance.

In the last two decades, proteomics has emerged as a powerful tool in biomarker discovery and mechanism understanding. Using these tools, researchers can efficiently perform large-scale screening to attain valuable information. Proteomics has been used as a tool to identify new disease related biomarkers in TNBC [11, 12]. Protein phosphorylation, one of the most ubiquitous posttranslational modifications (PTMs), is a key event in regulating many vital functions in cells including proliferation, survival, apoptosis, and signal transduction [13–15]. Protein phosphorylation, involved in signal transduction of the cells, requires a coherent activation of protein kinases and phosphatases, which leads to the defined functions [16]. The basal level of the phosphor-proteins may also represent the characters of the cells. For example, Stearns et al. [17] reported that the stable tyrosine phosphorylation of the IL-10 receptor may increase TIMP-1 levels to block tumor cell invasion in modified Boyden chamber invasion assays. Börner et al. [18] reported that the stable phosphorylation of the inhibitory Tyr-505 of the leukocyte-specific protein tyrosine kinase (Lck) may arrest Lck in its inhibited form. In recent years, the advances in phosphoproteomics research have allowed discovery of many important functions operating in cancer progression. Oyama et al. performed quantitative phosphoproteome and transcriptome analysis on ligand-stimulated MCF-7 breast cancer cells to study the mechanism of tamoxifen resistance [19]. They found that GSK3*β* and AP-1 transcription factors might be involved in the tamoxifen resistance in MCF-7 cells [19]. Rexer et al. used a phosphoproteomic approach to study lapatinib-resistance of HER2-overexpressing human breast cancer cell lines and found that the increased Src kinase activity was a mechanism of lapatinib resistance [20]. Oliveras-Ferraros et al. also reported a study on TNBC cell lines using low throughput phosphoproteomic approaches [21]. However, there has been no study focusing on dissecting TNBC drug resistance using large-scale phosphoproteomic tools. In this study, we used high throughput technologies to study changes in phosphorylated proteins to uncover important pathways involved in TNBC drug resistance.

For the purpose of this study, TNBC cell lines responding to multiple chemotherapeutic drugs were studied and were compared. Twelve established TNBC cell lines were tested against four chemodrugs and the half maximal inhibitory concentrations (IC50s) were calculated. The phosphorylated peptides of four resistant and two sensitive cell lines were analyzed using LC-MS/MS to discover important pathways related to chemodrug resistance of TNBC. Our study may lead to identification of useful prognostic biomarkers and therapeutic pathways for TNBC treatment.

## 2. Materials and Methods

### 2.1. Human Breast Cancer Cell Lines and Cell Culture

Triple negative breast cancer cell lines MDA-MB-231, MDA-MB-468, MDA-MB-436, HCC1187, HCC1806, and HCC1937 (all of which stain negative for ER, PR, and lack Her2/neu amplification) were obtained from American Tissue Type Culture Collection (ATCC, Manassas, VA, USA) [22, 23]. Cells were maintained in Dulbecco's minimal essential medium (Invitrogen, Carlsbad, CA, USA) or RPMI 1640 (Invitrogen) with 10% heat-inactivated Fetal Bovine Serum (Thermo Fisher Scientific, San Jose, CA, USA), 100 units/mL penicillin, and 100 *μ*g/mL streptomycin, at 37°C in 5% CO_2_.

### 2.2. In Vitro Drug Sensitivity Assay

Cell lines were treated with docetaxel, doxorubicin, gemcitabine, and cisplatin in vitro to determine the half maximal inhibitory concentration (IC50) of each drug. The cells were treated with DMSO or twenty predetermined doses of each drug for two days. Cell viabilities were determined by CellTiter-Glo Luminescent Cell Viability Assay (Promega, Madison, WI, USA). Triplicated experiments were performed twice and the IC50s were calculated using GraphPad Prism 5 software.

### 2.3. Sample Preparation

Cultured cells were lysed in lysing buffer (8 M urea, 4% CHAPS, 40 mM Tris-base, 65 mM DTT, 1% SDS) and the supernatant was collected into 1.5 mL tubes. Protein concentration of the lysate was determined using the Pierce BCA protein assay (Thermo Fisher Scientific). 20 *μ*L of 1 M DTT (Thermo Fisher Scientific) was added to samples containing 1 mg of proteins and incubated at 56°C for 1 hour and followed by an incubation at room temperature for 40 min in darkness with 80 *μ*L of 1 M IAA added into the buffer. Each sample was treated with 0.11 volumes of ice-cold 100% trichloroacetic acid (TCA) (Sigma Aldrich, St. Louis, MO, USA) on ice for 10 min and 500 *μ*L of ice-cold 10% TCA on ice for 20 min and then was spun down at 20,000 ×g for 30 min. The pellet was washed with 500 *μ*L of acetone and was centrifuged again at 20,000 ×g for 10 min. The protein pellets of all samples were collected and dried in a vacuum evaporator. 500 *μ*L of 100 mM ammonium bicarbonate (Sigma Aldrich) and 20 *μ*g of trypsin (Promega, San Luis Obispo, CA, USA) were added to the sample in each tube at 37°C for 2 hours. An additional 20 *μ*g of trypsin was added to the sample and subsequently incubated for 16 hours. The peptides were then filtered through 10 kDa filter columns (EMD Millipore, Billerica, MA, USA) and dried via vacuum evaporator.

### 2.4. Phosphopeptide Enrichment

400 *μ*L of loading buffer (80% acetonitrile (ACN) and 2% trifluoroacetic acid (TFA)) was added to 1 mg of peptides. The mixture was incubated with 4 mg of TiO_2_ beads (GL Sciences, Torrance, CA, USA) for 1 hour. The samples were centrifuged at 3,000 ×g for 5 min and the supernatant was discarded. TiO_2_ beads were collected and washed with 1 mL wash buffer I (30% ACN, 2% TFA) followed by 1 mL of wash buffer II (80% ACN, 0.1% TFA), each for 20 min at 4°C with rotation and centrifuged at 3,000 ×g for 5 min. The phosphopeptides were eluted first with 400 *μ*L elution buffer I (400 mM NH_4_OH, 50% ACN, pH 11) and was followed by 400 *μ*L elution buffer II (500 mM NH_4_OH, 60% ACN, pH 11). Nest Group MicroSpin strong cation exchange solid phase extraction tubes (The Nest Group Inc., Southborough, MA, USA) were used as a separation technique before mass spectrometry to reduce the complexity of samples and enhance the identification rate.

### 2.5. LC-MS/MS Analysis of Peptides

All peptide fractions were desalted before analysis using C18 tips made from the Empore C18 90 mm Disk (3 M Corporate, St. Paul, MN, USA). Nanoliquid chromatography and tandem mass spectrometry (nLC-MS/MS) with Collision Induced Dissociation (CID) was performed on a LTQ-Orbitrap (Thermo Fisher Scientific) integrated with an Eksigent nano-LC (Eksigent Technologies, Monmouth Junction, NJ, USA). The flow rate for reverse-phase chromatography was 500 nL/min for the loading and analytical separation (Buffer A: 0.1% formic acid, 3% ACN; Buffer B: 0.1% formic acid, 100% ACN). Peptides were resolved by the gradient of 3–40% buffer B over 180 min. The Orbitrap was operated in data-dependent mode with a full precursor scan at high-resolution (60000 at* m/z* 400) and ten MS/MS experiments at low resolution on the linear trap while the full scan was completed. For CID the intensity threshold was set to 5000 and the mass range was 350–2000.

### 2.6. Database Searching and Analysis

Mass spectra were searched against the Uniprot Human database using Proteome Discoverer software (Version 1.4, Thermo Fisher Sceintific), utilizing the Sequest (Thermo Fisher Scientific), Mascot (Matrix Science, London, UK), and X! Tandem (http://www.thegpm.org/tandem/) algorithms, while running a target decoy search strategy to increase protein identity confidence. Mascot and Sequest were searched with a fragment ion mass tolerance of 0.80 Da and a parent ion tolerance of 10.0 PPM and tolerated up to two missed trypsin cleavages. Carbamidomethylation of Cysteine was specified as a fixed modification. Glu or Gln to pyro-Glu of the n-terminus, oxidation of methionine, and phosphorylation of serine, threonine, and tyrosine were specified as variable modifications.

The Proteome Discoverer search results were loaded to Scaffold (Version 4.1.1, Proteome Software Inc., Portland, OR, USA) to quantify and validate the MS/MS peptide and protein identifications. Identifications were accepted if they had a greater than 95% peptide probability and contained at least one identifiable phosphopeptide. Scaffold PTM (Version 2.1.3, Proteome Software Inc.) was used to annotate phosphosites located in MS/MS spectra. Phosphosite localization probabilities were calculated using the Ascore probability based scoring technique [24] and only sites that met the stringent minimum of 99% were accepted.

The spectral count data of the phosphopeptides were acquired through Scaffold PTM and were compared between grouped cell lines: (1) all four resistant cell lines were compared with the two sensitive cell lines HCC1806 and MDA-MB-468; (2) resistant cell lines MDA231 and MDA-MB-436 were compared to the two sensitive cell lines; (3) resistant cell lines HCC1187 and HCC1937 were compared to the two sensitive cell lines. A *t*-test of each peptide between the groups of the three comparisons was performed and the peptides with *P* values < 0.05 and a fold change of at least 2.0 (≧2.0 or ≦0.5) were considered differential peptides. From the comparison (1), all changed phosphopeptides were further analyzed using online database String which is a database of known and predicted protein interactions including direct (physical) and indirect (functional) associations [25].

Hierarchical clustering analysis was performed using Euclidean distance formulation and complete linkage criteria for linkage of normalized phosphopeptide spectrum counts. Based on the comparison (1), only the peptide spectrum counts with *P* values less than 0.05 were imported to Permutmatrix software [26].

## 3. Results

### 3.1. Chemotherapy Drug Sensitivity Assay and Cell Line Selection

Protein phosphorylation is an important posttranslational modification that governs many of the signaling changes in cancer cells. The current study was performed to screen the signaling pathway changes, through the comparison of phosphorylated proteins in TNBC cell lines with extreme responses to the four chemotherapy drugs. The half maximal inhibitory concentrations (IC50s) of twelve TNBC cell lines against four chemotherapy drugs were determined and four resistant and two sensitive cell lines were selected for further analysis. Cell lines were ranked according to their IC50s against each drug (Table 1). No response is defined as the inability to reach a 50% of inhibition when the highest dose of chemotherapeutic drug was administered. Coefficient of Determination *R* ^2^ values of the drug sensitivity curves (data not shown) less than 0.7 were also considered as no response. HCC1187, MDA-MB-436, MDA-MB-231, and HCC1937 had the highest IC50s for at least three of the four drugs and were thus considered chemotherapy-resistant. HCC1806 and MDA-MB-468 were ranked lowest on the IC50 scale for at least three of the four drugs and were considered chemotherapy-sensitive. These six cell lines were selected for phosphorylated protein analysis.

### 3.2. Phosphopeptides Identification and Overall Results Profiling

**Figure 1:**
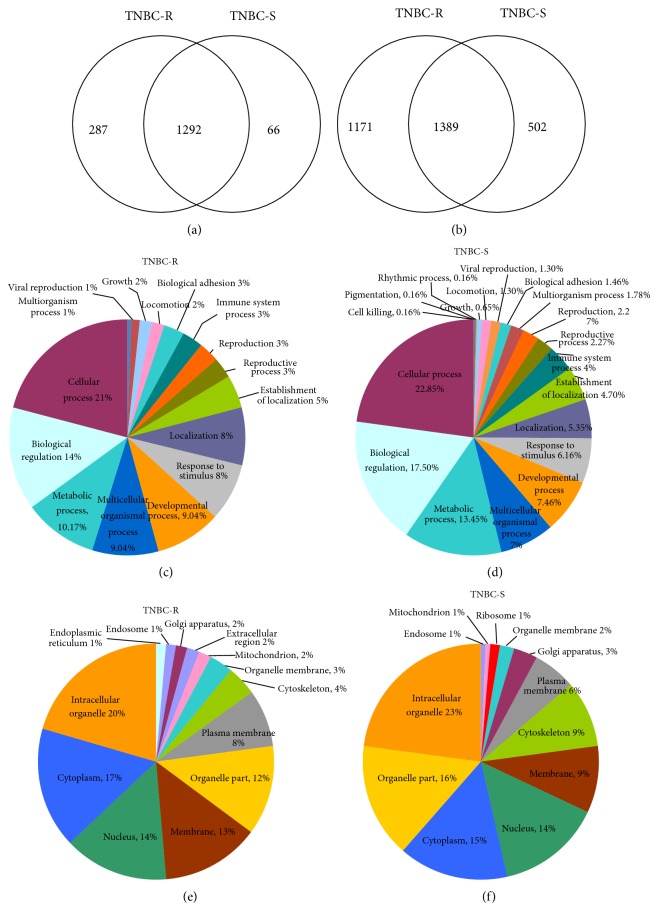
Phosphoproteins and phosphopeptides identified in TNBC. (a) Venn diagram of the total unique phosphoproteins identified in the two groups of cell lines (chemotherapy-resistant and chemotherapy-sensitive). 287 unique phosphoproteins were found only in resistant cell lines (TNBC-R) and 66 phosphoproteins were found only in sensitive cell lines (TNBC-S). (b) Venn diagram of the total unique phosphopeptides identified in the two groups of cell lines. 1171 unique phosphopeptides were found only in resistant cell lines and 502 phosphopeptides were found only in sensitive cell lines. Peptides with the same sequence, but different phosphorylated sites, were counted as one peptide in this diagram. (c) Pie graph illustrates the biological process of phosphoproteins only identified in resistant TNBC cell lines. (d) Pie graph illustrates the biological process of phosphoproteins only identified in sensitive TNBC cell lines. (e) Pie graph illustrates the cellular compartments of phosphoproteins only identified in resistant TNBC cell lines. (f) Pie graph illustrates the cellular compartments of phosphoproteins only identified in sensitive TNBC cell lines.

**Figure 2:**
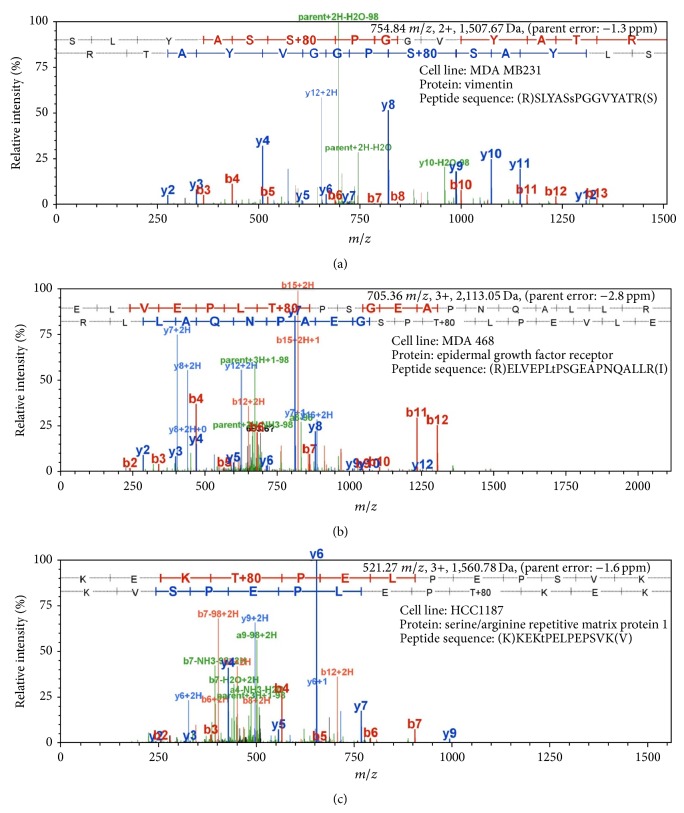
Representative MS/MS spectra for 3 phosphopeptides. (a) MS/MS coverage of vimentin in MDA-MB-231 cell line. Peptide sequence of “SLYASsPGGVYATR” and phosphorylated serine was found upregulated in the resistant cell lines. (b) MS/MS coverage of epidermal growth factor receptor in MDA-MB-468 cell line. Peptide sequence of “ELVEPLtPSGEAPNQALLR” and phosphorylated threonine was found downregulated in the resistant cell lines. (c) MS/MS coverage of serine/arginine repetitive matrix protein 1 in HCC 1187 cell line. Peptide sequence of “KEKtPELPEPSVK” and phosphorylated threonine was found downregulated in the resistant cell lines.

To increase the accuracy of identification, the spectra were cross-validated using three searching algorithms (Sequest, Mascot, and X! Tandem) against the Uniprot-Human database with the application of a decoy database. Proteome Discoverer was used in tandem with Scaffold and Scaffold PTM for quantification. A total of 1,645 phosphoprotein groups were identified by scaffold software across all six TNBC cell lines, using peptide probability thresholds of 95% with a minimum of one unique peptide (Figure 1(a)). The peptide False Discovery Rates (FDR) is 1.2% (Decoy) and protein FDR is 19.6%. All the decoy peptides and proteins were excluded. Accordingly, a total of 9,585 unique phosphopeptides with 10,091 unique phosphosites were identified (see Supplementary Table  1 in Supplementary Material available online at http://dx.doi.org/10.1155/2014/390781). Instances where the same peptide sequence had different phosphosites were counted as one peptide and ultimately 3,062 peptide sequences were identified (Figure 1(b)). The overall class profiles of the phosphoproteins specifically for resistant and sensitive cell lines were also expressed according to their gene ontology (GO) annotation. When compared to each other, more phosphoproteins involved in cellular adhesion processes were found in resistant cell lines while in sensitive cell lines more phosphoproteins were associated with multiorganism processes, cell killing, pigmentation, and rhythmic processes (Figures 1(c) and 1(d)). When the cellular compartments of these proteins were studied, the resistant cell lines showed more phosphoproteins in extracellular regions, mitochondrion, and plasma membrane whereas in sensitive cell lines more phosphoproteins were found in Golgi apparatus and cytoskeleton (Figures 1(e) and 1(f)).  Figure 2 shows the representative MS/MS spectra for three phosphopeptides marked with phosphosites. These phosphopeptides differed dramatically between drug resistant and sensitive cell lines and will be discussed below. The high quality spectra maps were very helpful in identifying peptides as well as phosphosites on them.

### 3.3. Phosphosites Confirmation and Label Free Quantification

In the current study, Scaffold PTM was used to further confirm the phosphosites identified and to construct a final quantification report. Scaffold PTM uses the Ascore algorithm to verify the presence of the phosphorylation sites. With the condition of a 99% Ascore certainty and 99% minimum localization probability threshold, a total of 1,340 phosphoproteins groups, 2,760 unique phosphopeptides, and 4,549 unique phosphosites were identified across all 6 cell lines (Supplementary Table  2). The mass spectra quantification data of these phosphopeptides were exported and further analyzed to determine the variations between chemotherapy-resistant and chemotherapy-sensitive cell lines (Supplementary Table  2). The spectrum counts of phosphorylated peptides were evaluated by computing the *P* value and fold change between the two groups of cell lines. Only phosphopeptides with a *P* value less than 0.05 and a fold change above 2 or less than 0.5 were considered. From three sets of comparisons (seen in Section 2), three sets of numbers were obtained (shown in Supplementary Table  3, Sheets 1, 2, and 3). All significantly changed phosphopeptides were further analyzed using the online String database for predicted protein interactions (see below).

### 3.4. Differential Phosphopeptides and Phosphoproteins Analysis

**Figure 3:**
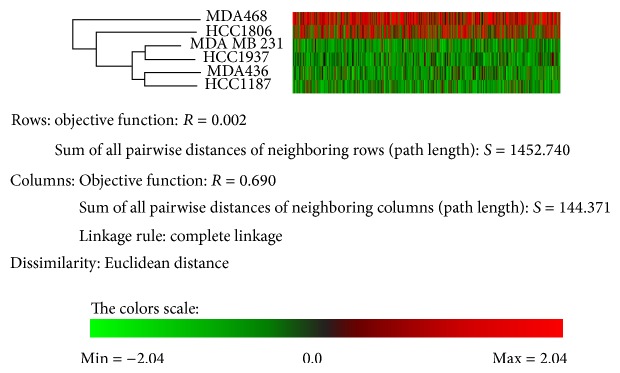
Phosphopeptides with differential abundance in TNBC cell lines. The hierarchical cluster displayed differential phosphopeptides between resistant and sensitive cell lines by Permutmatrix software. Only phosphopeptide changes with *P* values less than 0.05 were loaded for the analysis. Hierarchical clustering validated the IC50 data that helped classify these TNBC cell lines into two dissimilar groups: chemotherapy-sensitive (MDA-MB-468 and HCC1806) and chemotherapy-resistant (MDA-MB-436, MDA-MB-231, HCC1937, and HCC1187).

**Figure 4:**
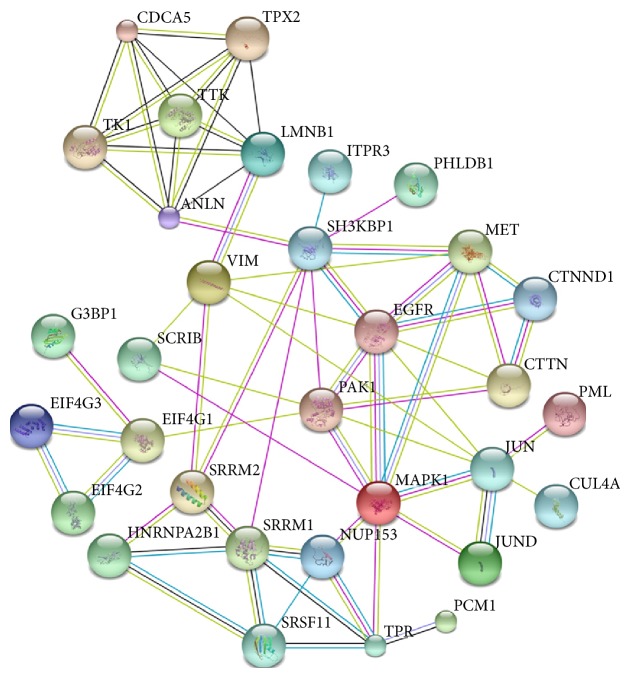
Protein network displaying associations between proteins found in the six TNBC cell lines using String database 9.05. Green lines represent neighborhood evidence; blue lines indicate cooccurrence evidence; purple lines indicate experimental evidence; light blue lines indicate database evidence; black lines indicate coexpression evidence.

**Figure 5:**
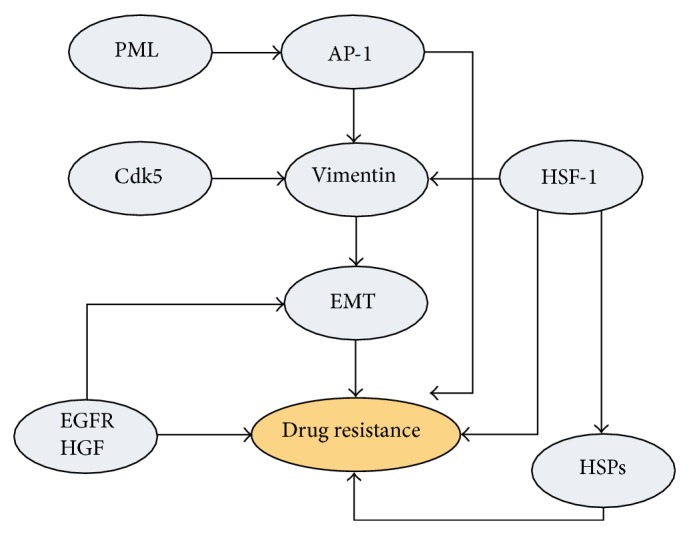
Overview of the pathways associated with altered phosphoproteins in the drug resistant of TNBC. PML, AP-1, and HSF-1 were shown to be activated in resistant TNBC cells and might promote downstream signaling including vimentin activation; activation of Cdk5 might also contribute to vimentin and LMNB1 activation to increase EMT in the resistant TNBC cells; EGFR and HGF in TNBC might contribute to the promotion of a multiple drug resistance (MDR) phenotype in resistant cells. Our data suggest that these signals work together to mediate the drug resistance of the TNBC cells.

Four drug resistant cell lines and two drug sensitive cell lines selected by IC50 ranking were subjected to phosphoproteome analysis in the current study. To confirm that these cell lines can be separated by the differentially expressed phosphopeptides into two groups: chemotherapy-resistant and chemotherapy-sensitive TNBC, the clustering analysis was performed.  Figure 3 shows that the four drug-resistant cell lines shared much more similarity with each other than the two sensitive cell lines and they could be clearly segregated from the two sensitive cell lines. This result further supported the IC50 data and suggested that the phosphopeptide identification and quantification methods are valuable in characterizing drug sensitivity of TNBC. The changed phosphopeptides described above were then analyzed with String database. All the changed phosphopeptides from Supplementary Table  3 were analyzed. The corresponding genes of these phosphopeptides were loaded to String to construct a network showing the associations between them. The genes with most connections in the network were shown in Figure 4 and Table 2. As shown in Figure 4, the changed phosphoproteins have strong associations and intricate interactions with one another. The pathways associated with the most prominent changes in drug-resistant TNBC are schematically summarized in Figure 5. These proteins and their roles in cancer drug resistance will be discussed.

## 4. Discussion

Over the last few decades there has been a steady decrease in breast cancer mortality rate largely due to the improvements in the treatment of breast cancer [27]. Despite the significant advancement made in breast cancer therapy in recent years, much of this progress is limited to hormone receptor positive and Her2/neu positive breast cancers. Specific targeted agents are still lacking for TNBC tumors leaving cytotoxic chemotherapy as the only therapeutic choice. Though cytotoxic chemotherapy is effective for many of these patients, the absence of a therapeutically targetable molecule or pathway limits the progress in treating these patients, which is manifested by rapid relapse and death when the tumors are resistant to the conventional chemotherapy. The identification of novel biomarkers indicative of drug sensitivity is imperative for the effective treatment of this exceptionally aggressive type of breast cancer. In our previous study we utilized a hydrophobic fractionation protocol in an effort to detect novel membrane proteins in triple negative breast cancer [28]. Although the identification of membrane biomarker is valuable, the heterogeneity of TNBC tumors commands the identification of a variety of biomarkers and signaling pathways in order to arrive at the best treatment strategy. It is important to identify aberrations in TNBC subtypes that cause these tumors to be resistant and unresponsive to chemotherapeutic treatments. Protein phosphorylation plays a crucial role in cellular signal transduction pathways. When activated, protein kinase binds and phosphorylates a specific substrate to mediate preprogrammed protein function [29, 30]. To find potential biomarkers that could predict the patient's response to chemotherapy and potential targets to improve TNBC treatment, we profiled phosphorylated peptides in TNBC cell lines with different sensitivities to a variety of chemotherapy drugs.

Label-free quantification was favored in this study since it has the largest dynamic range and highest proteome coverage-a prerequisite for the objective of this study as protein phosphorylation is a transient process and can be found with varying concentrations [31, 32]. Fractionation via SCX was also utilized in this study to decrease the sample complexity and thus improve the identification. Software as well as several computer-based algorithms was also used to correct any inconsistencies and to increase reliability, thus allowing for more accurate results. As shown in Figure 3, the phosphopeptide clustering data perfectly matched the drug sensitivity data (Table 1). These results gave additional proof that the quantification and identification system used was reliable and potentially had predictive value.

Because different phosphosites in a protein may trigger either protein activation or inactivation, we used phosphosite instead of phosphoprotein as unit for quantification. The functions of proteins will be discussed according to the changes of phosphosites. Among the dramatically altered phosphoproteins identified in the current study, many have also been reported to be important in tumor progression and/or drug resistance. Our results further support the roles of these proteins in TNBC drug resistance and offer new insights for future studies. Some new phosphorylated sites found in this study together with several of the previously reported protein phosphosites were connected for the first time to TNBC drug resistance. The important signaling changes are discussed below.

Transcription factor AP-1 (AP-1) is a multiprotein transcription factor and a member of the Jun and Fos protoontogenetic family. Previous studies have linked the activated AP-1 and its family members to increased tumorigenesis, metastasis, and invasion [33, 34] as well as drug resistance [35, 36]. Overexpression of phosphorylation at Ser73 of c-Jun was reported to be responsible for the development of multidrug resistance in colorectal cancer cells [37]. In the current study phosphorylated Ser73 of c-Jun was only found in the resistant cell lines, suggesting AP-1 is important in TNBC drug resistance. The overexpression and activation of promyelocytic leukemia protein (PML) is known to induce the transcriptional activation of AP-1 [38, 39]. In this study, we also found an upregulation of Ser583 on PML in drug resistant cell lines, a new phosphosite which has not been previously described. Our data suggests that this phosphosite provokes the activation of PML, which may lead to TNBC drug resistance through the activation of AP-1.

Heat Shock Factor 1 (HSF-1) is a master regulator for the transcription and heat shock proteins (HSPs) and was reported to induce a multidrug resistance phenotype through constitutive activation of the multidrug resistance gene 1 (MDR-1) [40]. In the current study phosphorylation of Ser303 on HSF-1 was identified to be upregulated in the resistant cell lines. Dai et al. proposed that phosphorylation of Ser303 induced a slow repression of HSF-1 allowing for the accumulation of the HSPs that are crucial for cell growth and recovery [41]. The above evidence suggested that Ser303 phosphorylation of HSF-1 might play an important role in TNBC drug resistance. In addition, both HSF-1 and AP-1 were capable of activating vimentin gene and could be responsible for its overexpression [42–44]. Vimentin is a well-recognized biomarker in epithelial to mesenchymal transition (EMT) and has been associated with metastasis, poorer prognosis, and cell motility in various types of cancer [45]. In the current study, Ser73 and Ser56 on vimentin were found to be phosphorylated in the resistant TNBC cell lines but were absent in the sensitive cell lines. Interestingly, cyclin-dependent kinase 5 (Cdk5) was reported to mediate phosphorylation at Ser56 of vimentin [46]. Ser23 of Lamin B1 (LMNB1) was also identified as an upregulated phosphosite in the resistant cell lines in this study. Others have shown that Ser23 is directly phosphorylated by Cdk5 as well [47]. Taken together, Cdk5 might be activated in drug resistant TNBC cell lines and might phosphorylate LMNB1 and vimentin, leading to EMT and drug resistance in TNBC.

It has been reported that epidermal growth factor receptor (EGFR) plays a significant role in hepatocyte growth factor receptor (HGF/c-Met) mediated biological activities [48]. Activation of HGF is indicative of aggressive tumor pathology, including enhanced proliferation and invasion [48]. EGFR might also promote the multiple drug resistance (MDR) phenotypes in breast cancer cells via accelerating the G1/S transition [49]. Furthermore, Bagowski et al. reported that EGFR phosphorylation at Thr654 and Thr669 by PRKD1 inhibits EGF-induced MAPK8/JNK1 activation [50]. Their studies showed that activation of EGFR and HGF might contribute to drug resistance of breast cancer through the downregulation of phosphosites such as Thr654 and Thr669 of EGFR. In the current study, the phosphosites Thr-693, Tyr-1197, and Tyr-1110 on EGFR and Thr977 on HGF were found to be downregulated in resistant cell lines, suggesting a release of negative regulation of EGFR and HGF functions in these cells. Downregulation of these phosphosites might increase EGFR and HGF activity and led to drug resistance of TNBC cell lines.

It is noticeable that many of the changed phosphoproteins found in the current study were involved in mRNA processing including heterogeneous nuclear ribonucleoprotein A2/B1, serine/arginine repetitive matrix 1 and matrix 2, eukaryotic translation initiation factor 4 gamma-1, and several others. As reviewed by Eblen, alternative splicing is a normal cellular process that can be manipulated by a cancer cell to enhance survival in response to chemotherapeutic treatment [51]. We also found the changes of phosphorylation status of some microtubule related proteins and DNA binding proteins in the current study (Figure 4 and Table 2). All these findings can be important in future TNBC drug resistance studies.

## 5. Conclusion

The current study has shown that proteomic analysis is a powerful tool for profiling of the phosphorylation patterns and may help better understand drug resistant TNBC cells. Our data suggested that PML, AP-1, and HSF-1 were preferentially activated in resistant TNBC cells and might promote downstream signals including vimentin activation. In addition, our study also suggested that Cdk5 might also promote vimentin and LMNB1 activation leading to an increased EMT in the resistant TNBC cells. We are also reporting on the potential roles of EGFR and HGF in the promotion of a multiple drug resistance (MDR) phenotype in TNBC. We have identified several signaling pathways that may work together to mediate the drug resistance in TNBC (Figure 5).

## Supplementary Material

Supplementary materials contain three Excel files with additional peptide data generated during this study. Table 1, total peptide and phosphosite list generated by Scaffold. Table 2, peptide quatification. Table 3, fold change data.

## Figures and Tables

**Table 1:** The IC50 ranking of twelve TNBC cell lines against four chemotherapy drugs.

IC50 ranking	Doxorubicin IC50 (nM)	Docetaxel IC50 (nM)	Gemcitabine IC50 (nM)	Cisplatin IC50 (nM)
1	HCC1395 (5783)	HCC1187 no response	HCC1187 no response	HCC1187 no response
2	MDA157 (1322)	MDA436 no response	MDA436 no response	MDA436 no response
3	HCC1937 (841.7)	HS578T no response	MDA231 no response	MDA231 no response
4	MDA436 (840.1)	HCC38 (318634)	HCC1395 no response	HCC1395 no response
5	MDA231 (644.6)	HCC1937 (253392)	MDA157 no response	MDA157 no response
6	HCC70 (531.4)	HCC70 (253375)	HS578T no response	HCC1937 (333854)
7	HS578T (454)	MDA231 (215645)	HCC1937 no response	HCC38 (232374)
8	HCC1187 (413.9)	HCC1395 (197207)	HCC70 no response	HCC1806 (208994)
9	BT20 (401.5)	MDA157 (160190)	BT20 (510.4)	BT20 (186719)
10	HCC1806 (233.2)	BT20 (149325)	MDA468 (146)	MDA468 (59710)
11	MDA468 (158.9)	MDA468 (2.378)	HCC38 (11.03)	HS578T (46238)
12	HCC38 (131.2)	HCC1806 (1.102)	HCC1806 (4.163)	HCC70 (30469)

**Table 2:** Selected phosphopeptide list with quantification data.

Protein name	Peptide	Gene	Site	231, 436, 1187, 1937 versus 1806 & 468		231 & 436 versus 1806 & 468		1187 & 1937 versus 1806 & 468	
				*P* value	Fold change	*P* value	Fold change	*P* value	Fold change
Serine/arginine repetitive matrix protein 1	KEKtPELPEPSVK	SRRM1	T220	0.001	0.39	0.023	0.35	0.049	0.42
Serine/arginine repetitive matrix protein 1	RYsPPIQR	SRRM1	S606	0.000	0.34	0.022	0.46	0.002	0.22
Serine/arginine repetitive matrix protein 1	RRsPsPPPTR	SRRM1	S558			0.038	Sensitive only		
Serine/arginine repetitive matrix protein 1	RRsPsPPPTR	SRRM1	S560			0.038	Sensitive only		
Catenin delta-1	VGGSsVDLHR	CTNND1	S269	0.005	Sensitive only	0.038	Sensitive only	0.038	Sensitive only
Catenin delta-1	GSLAsLDSLR	CTNND1	S349	0.008	0.31	0.013	0.08		
Catenin delta-1	GsLASLDSLR	CTNND1	S346	0.035	0.25	0.038	0.25		
Catenin delta-1	sGDLGDMEPLK	CTNND1	S920	0.035	0.25	0.038	0.25	0.038	0.25
Catenin delta-1	HYEDGYPGGSDNYGsLSR	CTNND1	S230					0.038	Resistant only
Sororin	IVAHAVEVPAVQsPR	CDCA5	S75	0.012	Sensitive only				
Eukaryotic translation initiation factor 4 gamma 2	FsPTMGR	EIF4G2	S395	0.011	0.39			0.005	0.11
Methyl-CpG-binding domain protein 1	VTNDIsPESSPGVGR	PCM1	S65	0.038	2.75	0.002	3.50		
Eukaryotic translation initiation factor 4 gamma 1	ANKtPLRPLDPTR	EIF4G1	T607	0.007	0.10			0.021	Sensitive only
Eukaryotic translation initiation factor 4 gamma 1	SFsKEVEER	EIF4G1	S1148					0.002	3.50
Eukaryotic translation initiation factor 4 gamma 3	RsPVPAQIAITVPK	EIF4G3	S99	0.043	3.75	0.033	4.0	0.015	3.50
Nuclear pore complex protein Nup153	sPGFASPK	NUP153	S645	0.005	Sensitive only	0.038	Sensitive only	0.038	Sensitive only
Alpha-2-HS-glycoprotein	cDSSPDsAEDVRK	AHSG	S138	0.033	7.33			0.002	11.67
Ras GTPase-activating protein-binding protein 1	SSsPAPADIAQTVQEDLR	G3BP1	S232	0.000	Sensitive only	0.013	Sensitive only	0.013	Sensitive only
Inositol 1,4,5-trisphosphate receptor type 3	VAsFSIPGSSSR	ITPR3	S1832	0.025	4.25	0.000	5.50		
Transcription factor AP-1	LAsPELER	JUN	S73	0.022	Resistant only	0.002	Resistant only		
Thymidine kinase, cytosolic	LFAPQQILQcsPAN	TK1	S231	0.000	Sensitive only	0.002	Sensitive only	0.002	Sensitive only
Thymidine kinase, cytosolic	scINLPTVLPGsPSK	TK1	S13	0.001	Sensitive only	0.020	Sensitive only	0.020	Sensitive only
Lamin-B1	AGGPTTPLsPTR	LMNB1	S23	0.025	Resistant only			0.013	Resistant only
Microtubule-associated protein 1B	VQSLEGEKLsPK	MAP1B	S1779	0.044	Resistant only	0.001	Resistant only		
Microtubule-associated protein 1B	SPSLSPSPPsPLEK	MAP1B	S1265			0.020	Resistant only		
Mitogen-activated protein kinase 1	VADPDHDHTGFLTEyVATR	MAPK1	Y187	0.043	0.21				
Phosphatidylserine synthase 2	DAGGPRPEsPVPAGR	PTDSS2	S16	0.004	Resistant only	0.020	Resistant only	0.002	Resistant only
Epidermal growth factor receptor	GSTAENAEyLR	EGFR	Y1197	0.000	Sensitive only	0.019	Sensitive only	0.019	Sensitive only
Epidermal growth factor receptor	RPAGSVQNPVyHNQPLNPAPSR	EGFR	Y1110	0.002	Sensitive only	0.030	Sensitive only	0.030	Sensitive only
Epidermal growth factor receptor	NGLQScPIKEDsFLQR	EGFR	S1064	0.006	Sensitive only				
Epidermal growth factor receptor	GSHQISLDNPDyQQDFFPK	EGFR	Y1172	0.008	Sensitive only				
Epidermal growth factor receptor	RPAGsVQNPVYHNQPLNPAPSR	EGFR	S1104	0.012	Sensitive only				
Epidermal growth factor receptor	mHLPsPTDSNFYR	EGFR	S991	0.039	Sensitive only				
Epidermal growth factor receptor	ELVEPLtPSGEAPNQALLR	EGFR	T693	0.003	0.16			0.025	0.13
Isoform 2 of hepatocyte growth factor receptor	VHtPHLDR	MET	T977	0.000	0.17	0.002	0.17	0.002	0.17
Heterogeneous nuclear ribonucleoproteins A2/B1	GGGGNFGPGPGsNFR	HNRNPA2B1	S225	0.000	Sensitive only	0.013	Sensitive only	0.013	Sensitive only
Heterogeneous nuclear ribonucleoproteins A2/B1	GGNFGFGDsR	HNRNPA2B1	S212	0.003	0.17			0.006	Sensitive only
Isoform PML-5 of protein PML	TPAsPHFR	PML	S583	0.025	Resistant only			0.006	Resistant only
Isoform 2 of dual specificity protein kinase TTK	VPVNLLNsPDcDVK	TTK	S240	0.035	Sensitive only				
PDZ and LIM domain protein 4	IHIDPEIQDGsPTTSR	PDLIM4	S112	0.031	8.00	0.009	12.00	0.037	4.0
Heat shock factor protein 1	VKEEPPsPPQSPR	HSF1	S303	0.026	Resistant only	0.038	Resistant only	0.021	Resistant only
Isoform 2 of serine/arginine-rich splicing factor 11	LNHVAAGLVsPSLK	SRSF11	S207			0.025	0.25		
Isoform 4 of acetyl-CoA carboxylase 1	SSmsGLHLVK	ACACA	S117	0.005	Resistant only	0.003	Resistant only	0.016	Resistant only
Serine/threonine-protein kinase PAK 1	sVIEPLPVTPTR	PAK1	S181	0.005	Sensitive only				
Beta-2-syntrophin	GPAGEAGAsPPVR	SNTB2	S110	0.034	2.31	0.002	3.25		
Beta-2-syntrophin	GLGPPsPPAPPR	SNTB2	S95			0.000	3.00		
Cullin-4A	KGsFSALVGR	CUL4A	S10	0.012	4.38			0.000	5.75
Isoform 2 of protein scribble homolog	ALsPAELR	SCRIB	S1405	0.029	Resistant only			0.005	Resistant only
Isoform 2 of protein scribble homolog	RVsLVGADDLR	SCRIB	S1297			0.005	0.33		
Isoform 2 of protein scribble homolog	NsLESISSIDR	SCRIB	S1139					0.021	3.50
Isoform 2 of protein scribble homolog	LPLLPPEsPGPLR	SCRIB	S772			0.019	0.41		
Src substrate cortactin	LQLHEsQKDYK	CTTN	Y421	0.035	Sensitive only				
Pleckstrin homology-like domain family B member 1	GGHERPPsPGLR	PHLDB1	S324	0.035	Resistant only			0.010	Resistant only
Isoform 2 of pleckstrin homology-like domain family B member 1	ALsPLPTR	PHLDB1	S470					0.005	0.14
Isoform 2 of actin filament-associated protein 1	SGTSSPQsPVFR	AFAP1	S752	0.013	Resistant only	0.006	Resistant only	0.037	Resistant only
Actin-binding protein anillin	AAsPPRPLLSNASATPVGR	ANLN	S182	0.000	0.12	0.004	0.24	0.000	Sensitive only
Myelin expression factor 2	AEVPGATGGDsPHLQPAEPPGEPR	MYEF2	S17	0.013	Resistant only	0.005	Resistant only	0.038	Resistant only
CAP-Gly domain-containing linker protein 2	KIsGTTALQEALK	CLIP2	S352	0.014	7.00				
Serine/arginine repetitive matrix protein 2	SSsPVTELASR	SRRM2	S1103	0.018	2.25			0.02	2.50
Serine/arginine repetitive matrix protein 2	MAPALSGANLTsPR	SRRM2	S2382	0.005	Sensitive only	0.049	Sensitive only	0.049	Sensitive only
Serine/arginine repetitive matrix protein 2	AGMSSNQSISsPVLDAVPR	SRRM2	S1404	0.035	Sensitive only				
Serine/arginine repetitive matrix protein 2	DIPRtPSR	SRRM2	T1472	0.035	Sensitive only				
Serine/arginine repetitive matrix protein 2	ScFESsPDPELK	SRRM2	S876					0.044	2.20
Serine/arginine repetitive matrix protein 2	ALPQtPRPR	SRRM2	T1492					0.02	0.50
Serine/arginine repetitive matrix protein 2	MALPPQEDATAsPPR	SRRM2	S1179	0.030	0.40				
Targeting protein for Xklp2	SSDQPLTVPVsPK	TPX2	S738	0.003	0.39	0.010	0.33	0.022	0.44
Nucleoprotein TPR	TDGFAEAIHsPQVAGVPR	TPR	S2155	0.028	0.50				
Vimentin	SLYASsPGGVYATR	VIM	S56	0.025	Resistant only	0.01	Resistant only		
Vimentin	LRSsVPGVR	VIM	S73	0.039	Resistant only	0.01	Resistant only		
